# Mapping the research trends and hot topics of ventricular arrhythmia: A bibliometric analysis from 2001 to 2020

**DOI:** 10.3389/fcvm.2022.856695

**Published:** 2022-10-20

**Authors:** Shiwei Wang, Tianyuan Jia, Guoxiang Liu, Xiaoye Lu, Qian Yang, Changqing Zhu

**Affiliations:** Department of Emergency Medicine, Renji Hospital, School of Medicine, Shanghai Jiao Tong University, Shanghai, China

**Keywords:** ventricular arrhythmia, bibliometric analysis, R package bibliometrix, CiteSpace, VOSviewer

## Abstract

**Background:**

Studies of ventricular arrhythmia (VA) have drawn much scholarly attention over the past two decades. Our study aimed to assess the current situation and detect the changing research trends of VA quantitatively and qualitatively.

**Materials and methods:**

All the information used in our statistical and bibliometric analysis were collected and summarized from papers retrieved from the Web of Science Core Collection (WoSCC) database on December 22, 2021 using certain criteria. Visual analytics were realized using CiteSpace, VOSviewer, the bibliometrix R package, and the bibliometric online analysis platform.

**Results:**

A total of 6,897 papers (6,711 original articles, 182 proceedings papers, three book chapters, and one data paper) were published in 796 journals that concentrated on the research areas of cardiovascular and critical care medicine. The most productive country and influential institution was the USA and the Mayo Clinic, respectively. *Heart Rhythm* (551 articles and 8,342 local citations) published the most manuscripts. The keyword co-occurrence and co-citation network of references analyses revealed that the most popular terms were ventricular tachycardia, ventricular fibrillation, catheter ablation, implantable cardioverter defibrillator (ICD), and sudden cardiac death (SCD). Further, the burst detection analysis demonstrated that topics strongly associated with clinical prognosis, such as meta-analysis, long-term outcomes, and impact, were new concerns.

**Conclusion:**

Our study offers a comprehensive picture of VA research and provides profound insights into the current research status. Moreover, we show that new topics within the VA research field have focused more on prognosis and evidence-based clinical guidelines.

## Introduction

The heart, the most vital organ of the human body, is a rhythmic pump that maintains the blood circulation system. Although its most fundamental anatomical structure and physiological functions were fully interpreted for the first time by William Harvey as early as 1628, our understanding of cardiac dysfunction and the prevention and palliation of related pathogenesis remains limited (1). Cardiovascular disease (CVD) is the leading cause of sudden cardiac death (SCD) worldwide, and in many cases, SCD is preceded by life-threatening cardiac arrhythmias, especially sustained ventricular arrhythmia (VA), which is frequently promoted by different types of CVD (e.g., acute coronary/non-coronary occlusion, left ventricular dysfunction, various types of cardiomyopathies, and primary electrophysiological abnormalities) (2–6). Even worse, the complexity of pathogenesis and heterogeneity of cardiac structural abnormalities have imposed tremendous challenges and additional difficulties for both the basic research domain of cardiac biology and clinical management and practice.

Fortunately, scientific and technological advances over recent decades have been rapid and we are no longer limited to anti-arrhythmic drug (AAD) therapy. The multiple new technologies and constantly updating clinical guidelines have helped obtain accurate diagnoses as well as prevent sudden death and recurrences of VA, thereby improving poor prognoses to some extent. Specifically, available and effective techniques for disease detection and treatment, such as 24-h ambulatory electrocardiogram (ECG) monitoring, pacemakers, implantable cardioverter defibrillators (ICDs), automated external defibrillators for cardiac arrest, and radiofrequency catheter ablation procedures, are all based on a better understanding of cardiac imaging in electrophysiology, myocardial biomechanics, and cardiac hemodynamics (1–3, 7). At the same time, many robust VA-related clinical guidelines, particularly those drafted and updated by the American Heart Association (AHA) and the Canadian Cardiovascular Society, have re-emphasized the great therapeutic value of ICDs and considered the cost-effectiveness of treatment decisions; they have also reiterated the importance of AAD medications as a crucial therapy for symptomatic treatment and the acute/long-term management of sustained VA in patients with structural heart disease (2, 8). Even so, extant research on VA and the relevant clinical guidelines for management still fall short, remain controversial, and require further detailed discussions. For instance, as stated by the AHA, there is a lack of evidence from highly reliable randomized controlled trials (RCTs) that AADs for VA improve survival when provided for the primary or secondary prevention of SCD, except for beta-blockers. Moreover, the overall incidence of specific AAD-induced arrhythmias, underlying mechanisms, and optimal methods to prevent other clinical side-effects and reduce risk remain unknown (2, 9).

Bibliometric analysis is frequently used to explore trends and hot topics in medical research fields, which enables the quantitative and visual measurement of research articles relating to countries/regions, institutions, journals, authors, and keyword networks (10). However, bibliometric studies of VA are scarce, with few comprehensive quantitative assessments of the status and trends of basic and clinical research. In this original research, by reviewing 6,897 papers published in 796 journals that concentrated on the research areas of cardiovascular and critical care medicine, we examined the existing circumstances and potential foci of the pertinent literature on VA published globally over the past 20 years. We hope that this original research will provide valuable insights and inspiration for future studies of VA.

## Materials and methods

### Data source and retrieval strategies

The Web of Science Core Collection (WoSCC), which is one of the largest bibliometric databases, was selected to collect the raw data to conduct our bibliometric analysis. To improve both precision and recall, the following search string was used within the document TITLE field: “TI = (Ventricular parasystole OR Ventricular *rhythmia* OR Ventricular tachycardia OR Ventricular fibrillation* OR Ventricular flutter OR Ventricular premature complex*)” with a time span from 2001 to 2020. The initial search returned 14,565 records. With the retrieval strategies refined by document type (article) and language (English), 6,953 records were finally obtained. All these article records were downloaded on the same day (December 22, 2021) to avoid the bias caused by frequent database updates.

### Data collection and extraction

Before being used for the descriptive analysis and bibliometric visualization, all the main information in our raw data (including article titles, authors and affiliations, countries, and journals) was converted into different txt formats according to the requirements of the software and then separately imported into CiteSpace (Drexel University, Philadelphia, United States; version 5.8.R3c SE), VOSviewer (Leiden University, Leiden, the Netherlands; version 1.6.17), a bibliometric online analysis platform,^1^ and RStudio (version 4.1.2) (11) using the bibliometrix R package.^2^ To ensure accuracy, the data collection and extraction processes were independently performed by two authors.

### Bibliometric analysis and science mapping

In the next step, we performed our bibliometric analysis in two stages. We first conducted descriptive statistics for the main document characteristics (annual number of publications, countries, affiliations, authors, and journals) and sorted them by the corresponding counts. To systematically and comprehensively analyze the overall trend of the research topic and of publications or citations, we relied on several frequently used evaluation metrics including Freeman’s betweenness centrality and the total/average number of article citations. In addition, to further evaluate the scientific impact of the most productive authors and journals, other indicators universally accepted by scholars, such as the h-index, m-index, quartile in category (Journal Citation Reports, JCR), and cited half-life (CHL), were introduced into the bibliometric evaluation system.

Additionally, to provide more comprehensive insights into highly cited documents in the field, we used the PlumX metrics (citations, usage, captures, mentions, and social media), derived from the Scopus database, to assess the impact of the research on social media. Citations reflect traditional measures of research impact; usage encompasses the counts of views, article downloads, library holdings, clicks, collaborators, and video plays; captures track the number of times an article is bookmarked, favorited, exported, and subscribed; mentions represent the number of citations in reviews, websites, topics, comments, and blogs; and social media show the frequency of likes, tweets, and shares across various platforms (12, 13).

Next, we performed bibliometric visualization and science mapping using a combination of the bibliometric online analysis platform and the other three types of software mentioned above to capture hidden patterns (conceptual, social, and intellectual network structures) and the dynamic evolution over time in the study area (14). Finally, we constructed collaboration network maps of countries, affiliations, and authors as well as carried out author keywords co-occurrence analysis, cited documents co-citation and cluster analysis, and burst detection analysis on the basis of the corresponding bibliographic records. For more details, parameter settings for CiteSpace are showed as follows: the number of years per slice was set to “1”; the selection criterion was set to “g-index”; and the scale factor k was set to “25”; furthermore, the options “pathfinder” and “non-pruning” were selected in order to retain the most salient structure of the network. Moreover, VOSviewer was used to create the term maps by the following options: “Creating a map based on bibliographic data,” “reading raw data from bibliographic database files,” “type of analysis: co-occurrence,” “unit of analysis: all authors keywords,” “counting method: full counting,” and “minimum number of the occurrence of a keyword: 20.” Definitions of some major statistical terms, metrics, and indices were summarized and provided in the Supplementary Table 1. Please see the Supplementary material for more details.

## Results

### Overview of the ventricular arrhythmia publications

Altogether, 6,897 documents comprising 6,711 articles, 182 proceedings papers, three book chapters, and one data paper were used for the bibliometric analysis based on our retrieval strategy and inclusion/exclusion criteria (time span from January 1, 2001 to December 31, 2020). Because different software programs use different counting and ranking algorithms, our ranking patterns showed subtle differences. Therefore, all the ranking metrics were identified using the combined results of the software and platform mentioned above. Table 1 summarizes the bibliometric statistics for the main data. The 6,897 documents related to VA research derived from 796 sources, covering 75,600 references and 24,358 authors. Additionally, 7,021 article keywords were reclassified and reclustered as “keywords plus” (6,645) based on the additional features of the WoSCC database. Moreover, the average number of citations per article was 24.12, which indicated that VA has been attracting significant research attention in the field of CVD. Furthermore, only 115 (0.47%) studies of VA were single-authored, while the remaining were multi-authored (24,243; 99.53%); the proportion of co-authors per article and collaboration index were 7.7 and 3.85%, respectively, suggesting that VA-related articles often require broad scientific collaboration.

**TABLE 1:** Summary of the bibliometric characteristics for articles in ventricular arrhythmia research.

Description for bibliometric characteristics	Results
Articles	6,897
Timespan	2001: 2020
Sources (Journals, Books, etc.)	796
References	75,600
Average citations per articles	24.12
Keywords Plus	6,645
Author’ s keywords	7,021
Authors	24,358
Author appearances	53,114
Authors of SA-articles	115
Authors of MA-articles	24,243
SA-articles	127
Articles per author	0.283
Co-Authors per article	7.7
Collaboration Index	3.58
**Article types**	
Article	6,711
Proceedings paper	182
Book chapter	3
Data paper	1

SA, single-authored; MA, multi-authored.

**FIGURE 1:**
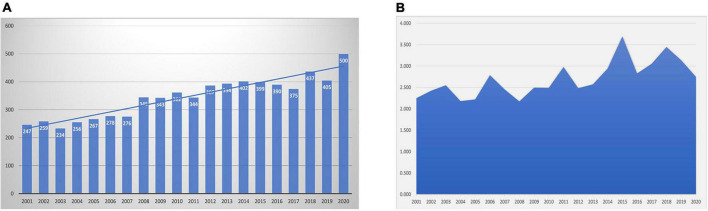
An overall view of publications in ventricular arrhythmia research field. Annual scientific publications of publications **(A)** and changing trends of annual average article citations **(B)** in the Web of Science Core Collection database, 2001-2020.

Figures 1A,B show the growth in annual publications and annual average citations for papers related to VA research from 2001 to 2020, respectively. Overall, a steadily increasing trend with only minor fluctuations was clearly apparent for annual publications. There were two turning points in the number of publications in 2008 and 2018 and a peak in 2020. Similarly, the average number of citations per year fluctuated from 2.172 (2008) to -3.693 (2015), peaking in 2003, 2006, 2011, 2015, and 2018; this might be due to the emergence of a large number of published and updated clinical guidelines.

### Distribution and collaboration of countries and affiliations

The sampled articles on VA stemmed from 72 countries and 4,786 affiliations. As shown in Table 2, the top 10 most productive countries and affiliations were identified by the total publications sorted by the corresponding author’s country and affiliation. Among the 72 countries, the United States contributed the greatest number of publications (7098), followed by China (2091), Japan (1887), Germany (1216), and Italy (1207). In addition, with regard to the betweenness centrality in this social network, we found that the United States (centrality = 0.90) again had the highest value, demonstrating that it was the most influential country, far exceeding the United Kingdom (0.08) and Canada (0.07).

**TABLE 2:** The top 10 most active countries and affiliations contributing to scientific production in ventricular arrhythmia (ordered by articles count).

Rank	Countries	Articles	Centrality	TC	AC	Affiliations	Articles	Centrality	TC	AC
1	USA	7,098	0.90	72,862	31.61	Mayo Clin	295	0.07	1,560	5.29
2	China	2,091	0.03	5,975	8.50	Univ Calif Los Angeles	207	0.11	1,792	8.66
3	Japan	1,887	0.01	11,816	16.90	Harvard Univ	194	0.08	1,760	9.07
4	Germany	1,216	0.05	7,893	21.68	Johns Hopkins Univ	146	0.04	899	6.16
5	Italy	1,207	0.05	11,386	36.85	Leiden Univ	145	0.07	1,432	9.88
6	France	1,021	0.06	6,583	32.11	Univ Michigan	137	0.04	1,390	10.15
7	Canada	808	0.07	7,248	33.56	Brigham and Womens Hosp	135	0.12	1,887	13.98
8	Netherlands	808	0.04	8,150	32.09	Natl Yang Ming Univ	133	0.02	312	2.35
9	England	730	0.08	5,697	23.44	Univ Alabama Birmingham	129	0.02	1,061	8.22
10	Spain	674	0.04	3,308	19.69	Univ Penn	128	0.04	1,038	8.11

TC, total number of article citations; AC, average number of article citations.

**FIGURE 2:**
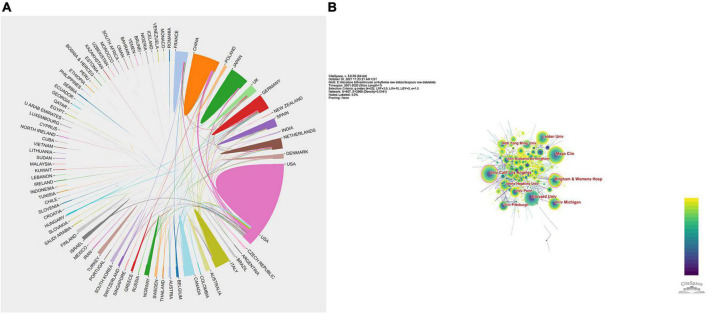
Cooperative relationship and intensity among authors’ countries and affiliations. Cooperation network map of collaborating countries **(A)** and affiliations **(B)** for ventricular arrhythmia research.

The visual network analysis showed the collaborations among countries, identifying that the most frequent cooperation appeared between the United States and China, followed by the United States and Japan (Figure 2A), which validated the significance of the centrality metric. Among the 4,786 affiliations, the Mayo Clinic topped the list (295), while Brigham and Women’s Hospital had a relatively high influence (centrality = 0.12), followed by the University of California Los Angeles (0.11) and Harvard University (0.07). Similarly, Figure 2B, drawn by CiteSpace, presents the collaboration network of affiliations involved in all 6,897 publications on VA research, showing a low distribution density (0.0161) and a centrality of the top 10 affiliations below 0.15 (Table 2). These results indicate that the distribution is relatively dispersed and that further academic collaborations are needed.

### Authors’ contributions to ventricular arrhythmia research

**FIGURE 3:**
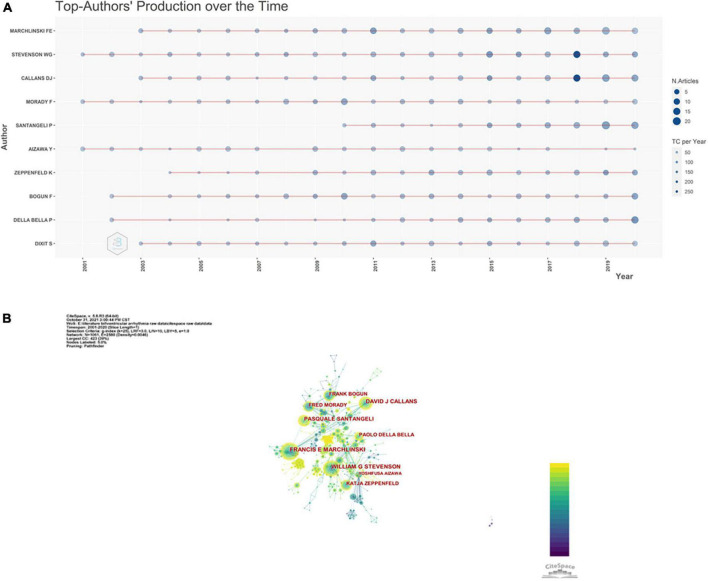
Distribution of author contributions to ventricular arrhythmia research. Top 10 authors’ production over time **(A)** and cooperation network map of collaborating authors **(B)**.

Table 3 shows the top 10 most productive of the 24,358 authors in the past 20 years, ranked by the number of publications. In addition to productivity (represented by the number of articles published by an author), the academic impact (represented by the average number of local citations) cannot be underestimated and should be considered when evaluating an author’s contributions to and relevance in a field. Taking all these conditions together, the top three best-performing authors, Francis E. Machlinski (123 articles; 1,997 citations), William G. Stevenson (111 articles; 2,029 citations), and David J. Callans (103 articles; 1,438 citations), were the most productive authors with a high average number of local citations and high contribution rate of authors to publications. Moreover, William G. Stevenson can be seen as an influential scholar on this topic, as reflected by an uninterrupted series of publications over the past two decades (Figure 3A). Interestingly, Fred Morady was the only author to publish VA-related studies as neither the first author nor the corresponding author, but still exhibited relatively high productivity and influence.

**TABLE 3:** The top 10 most relevant authors that published articles on ventricular arrhythmia research (ordered by articles count).

Rank	Author	Articles	AFF	Centrality	TLC	ALC	First author counts	First author citations	Average first author citations	Corresponding author counts	h-index	m-index
1	Marchlinski, FE	123	15.20	0.01	1,997	16.24	2	86	43.00	44	42	2.21
2	Stevenson, WG	111	14.93	0.03	2,029	18.28	5	281	56.20	29	41	1.95
3	Callans, DJ	103	10.92	0.00	1,438	13.96	1	5	5.00	6	38	2.00
4	Morady, F	80	9.59	0.01	1,242	15.53	0	0	0.00	0	32	1.52
5	Santangeli, P	79	6.10	0.00	552	6.99	12	166	13.83	14	24	2.00
6	Aizawa, Y	68	8.25	0.00	251	3.69	9	47	5.22	17	18	0.86
7	Zeppenfeld, K	68	8.43	0.05	737	10.84	3	69	23.00	34	27	1.50
8	Bogun, F	67	7.69	0.01	1,080	16.12	15	363	24.20	62	29	1.45
9	Della Bella, P	67	6.59	0.01	756	11.28	9	192	21.33	24	27	1.35
10	Dixit, S	67	6.49	0.00	1,132	16.90	3	91	30.33	10	31	1.63

TLC, total number of local citations; ALC, average number of local citations; AFF, articles fractionalized frequency.

As described in “Materials and methods” section, to better assess the scientific impact and relevance of the community and avoid bias, other well-established indicators were introduced into our bibliometric analysis. Table 3 also lists the h-index and m-index of the top 10 most productive authors. The overwhelming majority of these authors had an excellent combination of productivity and influence, apart from Yoshifusa Aizawa, who had published 68 articles but only received 251 local citations with an average number of local citations of 3.69, a local h-index of 18, and an m-index of 0.86. The m-index, calculated from the h-index weighted for an author’s career duration in years, is considered to be a highly representative measure of an individual’s scientific achievement (15). More interestingly, except for the top three best-performing authors mentioned above, Pasquale Santangeli is extremely competitive and highly influential on this topic despite not having published papers in this field until 2010 (Figure 3A and Table 3). Similar to above, Figure 3B shows the authors’ collaboration networks in VA research, again highlighting that better networking is necessary to facilitate domestic and international collaborations and co-authorships across academia.

### Journal distribution

**FIGURE 4:**
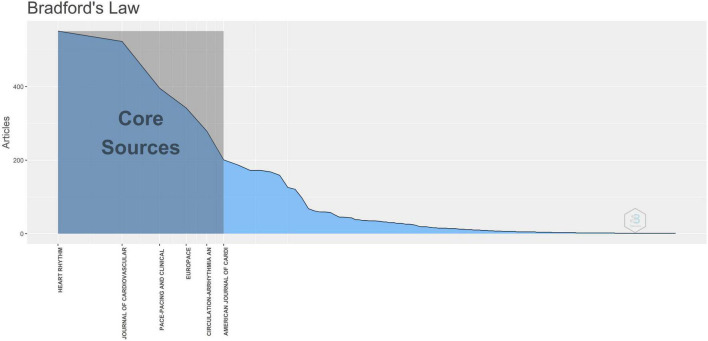
Journal distribution of publications in ventricular arrhythmia research according to Bradford’s law.

As mentioned above, 796 journals published 6,897 articles in this field over the sampled 20 years. Figure 4 shows that the distribution of the most important journals in terms of contributions was in accordance with Bradford’s literature dispersion law (16). To characterize these high-impact journals more representatively, Table 4 lists the top 10, which published 2,823 articles (accounting for 40.93% of all the publications in our bibliometric study). As expected, most VA-related studies were published in the most prestigious and highest-ranking journals in the field of CVD, while only one journal (*Resuscitation* tied with *Journal of the American College of Cardiology* in eighth place) belonged to the emergency and critical care medicine area. Further, the top three journals published more than half of the papers listed in this table. However, in terms of local citations received, the advantage of the top three journals diminished compared with *Circulation* and the *Journal of the American College of Cardiology*, validating the need for other evaluation metrics. Therefore, after comprehensive consideration, both productivity and influence coupled well in *Circulation*, the *Journal of the American College of Cardiology*, and *Heart Rhythm*, which was reflected not only by the higher impact factors, but also by the appreciable h- and m-indices (see Table 4 for more details). Furthermore, the m-index of *Circulation: Arrhythmia and Electrophysiology* (one of the *Circulation* series) was far ahead of its counterparts in this area, which presents great output potential for scientific research on VA. Additionally, combined with the data provided by the Journal Citation Reports assessment system and bibliometric online analysis platform, our research results imply that the top 10 journals are mainly concentrated in Q1 and Q2, which indicates that most are highly influential in the relevant domains.

**TABLE 4:** The top 10 most productive journals that published articles on ventricular arrhythmia research (ordered by articles count).

Rank	Journal title	Countries	Articles	TLC	ALC	IF (2020)	h-index	m-index	Quartile in category (2020 JCR)	Cited Half-life
1	Heart Rhythm	USA	551	8,342	15.14	6.343	65	3.61	Q1	5.8
2	Journal of Cardiovascular Electrophysiology	USA	523	8,222	15.72	2.871	50	3.25	Q2	8.2
3	Pace-pacing and Clinical Electrophysiology	USA	396	4,584	11.58	1.976	29	1.38	Q3	10.4
4	Europace	England	342	3,066	8.96	5.214	33	1.57	Q1	5.0
5	Circulation-arrhythmia and Electrophysiology	USA	279	4,444	15.93	6.572	56	4.00	Q1	6.0
6	American Journal of Cardiology	USA	201	6,787	33.77	2.778	37	1.76	Q2	11.9
7	Circulation	USA	187	26,707	142.82	29.690	85	4.05	Q1	11.1
8	Journal of the American College of Cardiology	USA	172	16,000	93.02	24.093	80	3.81	Q1	7.6
9	Resuscitation	Ireland	172	2,677	15.56	5.262	37	1.76	Q1	6.1
10	Journal of Interventional Cardiac Electrophysiology	Netherlands	168	945	5.63	1.900	17	0.90	Q3	5.0

TLC, total number of local citations; ALC, average number of local citations; IF, impact factors.

The CHL of these journals refers to the time (in years) required for the articles to reach nearly half of the current citations received. Usually, a higher CHL value for a journal suggests a higher citation frequency of papers previously published in this journal than those published recently. The vast majority of the top 10 most productive journals scored relatively high CHL values (above 5); among these, the CHL values of *Pacing and Clinical Electrophysiology*, the *American Journal of Cardiology*, and *Circulation* exceeded 10. These results indicate that additional and recent high-quality studies are constantly needed for future scientific activities and clinical research.

### Highly cited articles

Table 5 lists the characteristics of the top 10 most globally cited articles, including their titles, first authors, journals, publication years, number of global/local citations, number of citations per year, and detailed PlumX metrics (obtained from the Scopus database). The predominant study types of these documents were clinical trials and related guidelines published in journals, especially *Circulation*, while animal experiments occupied a certain proportion. Table 5 also shows that Silvia G. Priori, who published fewer relevant articles on this topic than those authors listed in Table 4, paid more attention to high-quality clinical trials and the potential for clinical translation; further, when he or she was the first author, the total number of global citations was at its peak. However, in addition to the two clinical guidelines drafted and released in 2015, the publication dates of all the remaining highly cited articles in *Circulation* were earlier than 2010, again verifying the efficiency of the CHL for evaluating the rate at which a journal ages.

**TABLE 5:** The top 10 most global popular articles and their bibliometric parameters (ordered by the number of global citations).

Rank	Article titles	Authors	Journal	Publication years	GC	LC	TC/Y	PlumX metrics				
								Citations	Usage	Captures	Mentions	Social Media
1	“2015 ESC Guidelines for the management of patients with ventricular arrhythmias and the prevention of sudden cardiac death The Task Force for the Management of Patients with Ventricular Arrhythmias and the Prevention of Sudden Cardiac Death of the European Society of Cardiology (ESC) Endorsed by: Association for European Paediatric and Congenital Cardiology (AEPC)”	Priori, SG	European Heart Journal	2015	1,220	91	174.29	1,985	67	1,346	10	15
2	“Adverse effect of ventricular pacing on heart failure and atrial fibrillation among patients with normal baseline QRS duration in a clinical trial of pacemaker therapy for sinus node dysfunction”	Sweeney, MO	Circulation	2003	999	30	52.58	1,204	9	279	1	51
3	“Mutations in the cardiac ryanodine receptor gene (hRyR2) underlie catecholaminergic polymorphic ventricular tachycardia”	Priori, SG	Circulation	2001	902	154	42.95	597	7	135	1	0
4	“ACC/AHA/ESC 2006 Guidelines for Management of Patients With Ventricular Arrhythmias and the Prevention of Sudden Cardiac Death A Report of the American College of Cardiology/American Heart Association Task Force and the European Society of Cardiology Committee for Practice Guidelines (Writing Committee to Develop Guidelines for Management of Patients With Ventricular Arrhythmias and the Prevention of Sudden Cardiac Death) Developed in Collaboration With the European Heart Rhythm Association and the Heart Rhythm Society”	Zipes, DP	Circulation	2006	811	106	50.69	936	188	358	4	2
5	“2015 ESC Guidelines for the management of patients with ventricular arrhythmias and the prevention of sudden cardiac death”	Priori, SG	Europace	2015	796	74	113.71	459	39	239	6	0
6	“Clinical and molecular characterization of patients with catecholaminergic polymorphic ventricular tachycardia”	Priori, SG	Circulation	2002	717	170	35.85	912	1	352	0	0
7	“Infarct tissue heterogeneity by magnetic resonance imaging identifies enhanced cardiac arrhythmia susceptibility in patients with left ventricular dysfunction”	Schmidt, A	Circulation	2007	551	84	36.73	609	91	336	0	0
8	“Effects of angiotensin-converting enzyme inhibition on the development of the atrial fibrillation substrate in dogs with ventricular tachypacing-induced congestive heart failure”	Li, DS	Circulation	2001	534	14	25.43	597	10	133	0	0
9	“Chest Compression Fraction Determines Survival in Patients with Out-of-Hospital Ventricular Fibrillation”	Christenson, J	Circulation	2009	518	6	39.85	551	3	314	4	166
10	“Adverse hemodynamic effects of interrupting chest compressions for rescue breathing during cardiopulmonary resuscitation for ventricular fibrillation cardiac arrest”	Berg, RA	Circulation	2001	500	22	23.81	569	498	295	0	0

LC, the number of local citations; GC, the number of global citations; TC/Y, the number of total citations per year.

Next, we used the PlumX metrics to offer a novel perspective in our bibliometric study. Although some differences were found in the results for the citations owing to the different counting types and algorithms in the different databases, the trends of the citations were still similar between the outcomes. However, most yielded low scores for usage and captures, particularly the former. Aside from the multicenter study by Jim Christenson et al., clinical trial by Michael O. Sweeney, and clinical guideline by Silvia G. Priori et al., in terms of mentions and social media, the number of citations received by the rest was very low, which was an unexpected finding.

### Keyword co-occurrence and analysis

**FIGURE 5:**
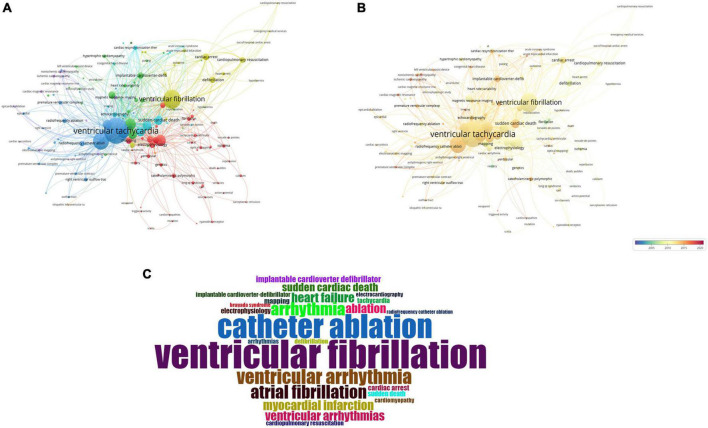
Co-occurrence and cluster analysis of author keywords. Network view map of author keywords co-occurrence **(A)**, the form of time distribution **(B)**, and word cloud chat **(C)**.

VOSviewer was used to create a distinct and intuitive network map using a co-occurrence analysis based on the article keywords in publications on VA research. Each sphere gizmo represented a keyword or key phrase whose size and color separately indicated the frequency of its appearance (recurrence in the same article counted as one) and average number of citations. In addition, the distance and degree of thickness of the lines between two sphere gizmos implied a more complex co-occurrence relation and vastly diverse link strength, respectively. As illustrated in Figure 5A, of the 7,021 samples, 165 keywords were finally screened out based on a threshold of at least 20 occurrences and then classified into seven clusters. Additionally, Figure 5B shows the change in these keywords over time, where the scale bar at the bottom right shows the color gradient indicating the different years. To make the analysis results more intuitive and visual, the hierarchical “word cloud” constructed using the specific R package was then introduced (Figure 5C).

### Reference co-citation and cluster network analysis

**FIGURE 6:**
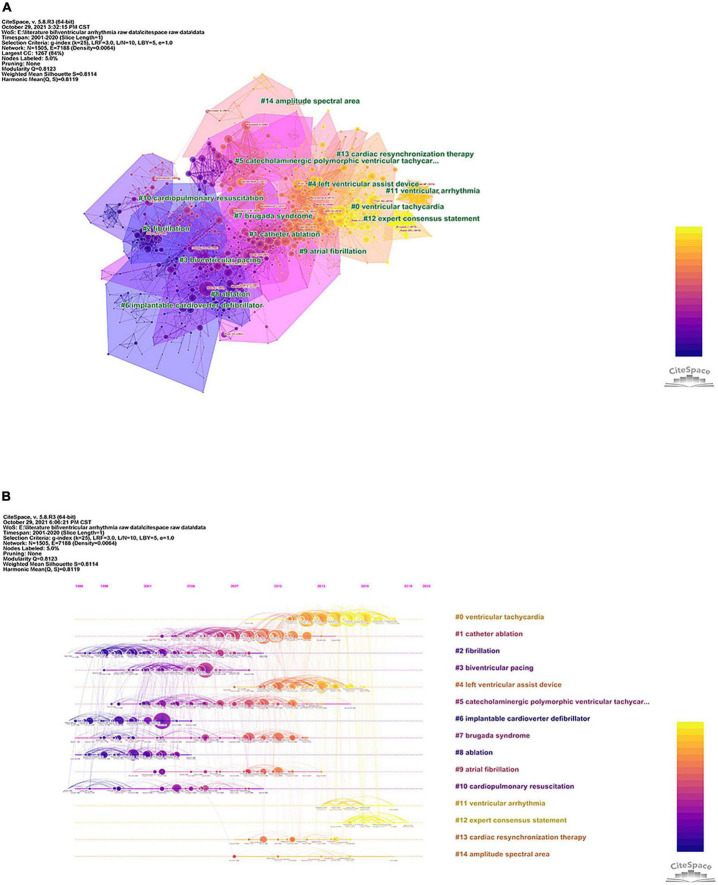
References’ co-citation and cluster analysis. Network map of co-cited references on ventricular arrhythmia research **(A)** and the timeline view with corresponding cluster labels on the right side **(B)**.

To further explore hot topics in VA-related research, a co-citation network map of the top co-cited references (except duplicates) and its other manifestation (the same network in a hierarchical order based on time) were created using CiteSpace (Figures 6A,B). The different clusters in Figure 6A are represented by different background colors and the size of each cluster is formed by different node types, indicating the number and centrality of the co-cited articles. Similarly, in Figure 6B, each node position indicates the time of co-citation and the size is proportional to the number of co-citations of the relevant reference. Finally, all the co-cited references were clustered into 15 major labels, including ventricular tachycardia (VT), catheter ablation, fibrillation, biventricular pacing, and left ventricular assist device. Figure 6B also shows a much more distinct co-citation network of references in a timeline view, clearly illustrating the higher degrees of citation bursts in cluster labels at different time points (e.g., #0, #1, #3, and #6).

### Burst detection analysis

**FIGURE 7:**
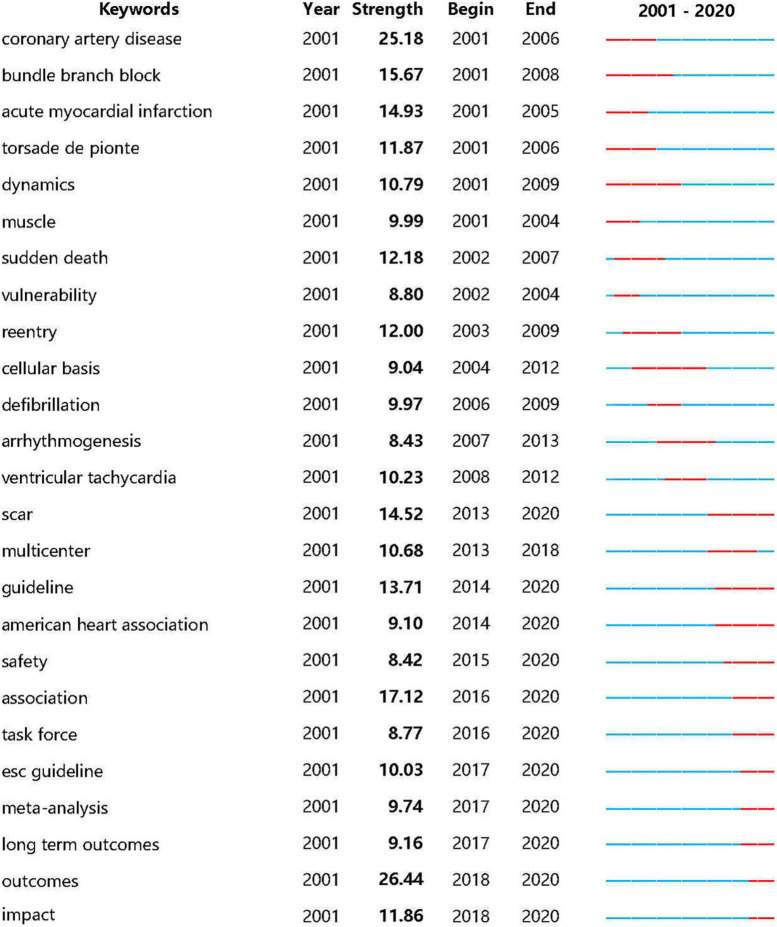
Burst detection with keywords in publications on ventricular arrhythmia research (only the top 25 keywords with the strongest citation bursts were shown).

Burst detection analysis, a computational technique for detecting sudden changes in events and other types of information, can be used to identify emerging concepts and themes that have attracted considerable attention in a field (17). The sky-blue line represents the time interval during 2001 to 2020, while the durations of each burst are plotted by the red line. Figure 7 shows the 25 keywords with the strongest citation bursts based on the consideration of research or clinical significance, indicating the evolution of VA research over the past two decades. Outcomes ranked first with the highest burst strength (26.44), followed by coronary artery disease (25.18), association (17.12), bundle branch block (15.67), and acute myocardial infarction (14.93). Moreover, there was a significant turning point in VA-associated research around 2012, when a sudden shift from pathogenesis to clinical practice occurred (see the “Discussion” section for more details).

## Discussion

In this original research, we conducted a bibliometric analysis of 6,897 publications on VA research from 2001 to 2020 using the visualization and statistical functions of the WoSCC database, a bibliometric online analysis platform, the bibliometrix R package, CiteSpace, and VOSviewer to identify the present situation and recent trends in research in this field. We found that the number of VA-related publications has steadily increased over the past two decades and peaked in the most recent year. The overwhelming contributions to these manuscripts were made by authors’ countries with highly economic and medical developments: By country, the United States ranked first, followed by China and Japan. Europe and the United States dominated the list of the top 10 most productive countries for VA publications, with only two from Asia. Similarly, the distribution of affiliations was concentrated in Europe and North America except for the National Yang Ming University from the Taiwan Province of China; imbalances in the development and distribution of medical resources account for this difference. Furthermore, the origin and consistent development of modern arrhythmia-related research and technology use, including the proposal of the early terminology and doctrine, “era of Einthoven and Thomas Lewis” maturation of electrocardiography, and establishment of cardiomyocyte electrophysiology, have mainly been attributed to collaborations among the authors, countries, and institutions in the Western world (1, 5, 18, 19).

In addition, combining the outcomes of the journal distribution analysis and the analysis of highly cited articles in the field clearly showed that *Circulation’s* status as the leading journal in the field is well deserved based on its number of publications on VA as well as their substantial influence. However, as shown by the CHL values and publication years, the vast majority of the most-cited papers in these journals was used as fundamental and background knowledge for the citing articles. Furthermore, despite animal experiments and clinical investigations representing a considerable proportion of the top 10 most-cited articles, all related academic outcomes were published before 2010. This illustrates that the study of VA has evolved to a relatively mature stage and that more creative and innovative ideas and study designs are essential for the future evolution of this active field of scientific research.

Another important factor influencing the scholarly impact of these studies is the authoritative guidelines in this field, including the origin and development of techniques for advancing VA treatment mentioned above as well as the updates and summaries of extraordinary authoritative clinical guidelines drafted and revised since the mid-1980s by the AHA, the American College of Cardiology, and the European Society of Cardiology, which account for the imbalanced distribution of high-impact medical journals and publications (2). Our findings were unexpected but reasonable in terms of the PlumX metrics. We did not expect to find the extraordinarily low number of social media use and records in the Scopus database for highly cited publications (specifically the mentions and social media metrics in Table 5). In the conventional sense, clinical guidelines are more likely to be shared on social media platforms and users than basic medicine experiments. Obviously, this is not the case. A major reason for this is the time to publication. Despite continuous and rapid advances in online social media over the past 20 years, it is difficult to keep pace with the medical literature due to the specific characteristics of academic papers such as their timeliness and fast update cycles. Moreover, academic researchers’ information management capability and familiarity with modern network techniques vary from person to person. The recent study by Ortega showed that journals with their own Twitter accounts often receive more tweets and higher citation frequencies than those without. This study made an interesting attempt to explore how the dissemination of content in an online social network influences the citation of research paper in their corresponding journals (20). Therefore, it is tempting to speculate that the PlumX metrics indeed provide a clear competitive advantage to those scientists active on social platforms by increasing the impact and dissemination of their research to the public (13, 20–22). Moreover, this inference was also proven by Christenson and his scientific research team, whose study ranked ninth in Table 5 with 166 citations on social media. In summary, to enhance their scientific influence and academic performance for different audiences, scholars need to broaden their horizons and make progress over time.

Next, our analysis of research hot topics and prediction of future trends using keyword co-occurrence analysis, co-citation network analysis, and burst detection analysis showed the central topics of the investigation of VA over time. These topics included pathogenesis/mechanisms (e.g., ventricular/atrial premature contraction/tachycardia/fibrillation, reentry, bundle branch block, and hypokalemia), the diagnosis of primary diseases/etiologies (e.g., coronary artery disease, Brugada syndrome, acute myocardial infarction, and cardiomyopathy), common arrhythmia-associated complications (e.g., sudden cardiac arrest, heart failure, and SCD), therapeutic technological innovations [e.g., radiofrequency catheter ablation (RFCA), left ventricular assist device, and implant cardiac resynchronization therapy defibrillator (CRT-D)], authoritative evidence-based guidelines and expert consensus statements (e.g., the AHA, the European Society of Cardiology, multicenter and meta-analysis), and prognostic assessment and prediction [short/long term outcomes, amplitude spectral area (AMSA), and impact]. Because of the diversity of etiologies and complexity and continuity of pathophysiological processes in VA, the above keyword results should be summarized and generalized. Moreover, VA is affected by a variety of factors, and it eventually develops a more severe form and a poor prognosis if timely interventions are not performed (i.e., malignant arrhythmias such as VT, ventricular fibrillation [VF], and even SCD) (3, 4, 6). Furthermore, as reported in our recent bibliometric study for cardiopulmonary resuscitation (CPR), although a great amount of research and constantly updated guidelines and therapeutic measures have focused on SCD secondary to VF and the continuous exploration of effective means to improve prognosis, overall survival from SCD remains low (23). Nevertheless, we cannot ignore the achievements already been made. For instance, based on the results above, AMSA, one of the cluster labels (shown in Figures 6A,B, #14), was also extracted from the co-cited references. Briefly, AMSA, whose algorithm was suggested, quantitated, determined, and gradually refined by Charles G. Brown et al. over the past 25 years, is considered as a potential and practical prognosticator for measuring the probability of successful defibrillation and guiding the optimal timing of defibrillation, which has been studied in clinical trials and large animal experiments. In addition, a large number of researchers are convinced that AMSA is suitable for clinical transformation, although many questions and challenges remain (24–27).

The response of any treatment intervention is likely to be limited in the advanced disease stage. By contrast, the early detection and intervention of arrhythmogenesis and aggressive prevention of SCD have become research foci and hot topics in the field. As ECG technology matures, various types of VAs can be quickly identified and precisely diagnosed at an earlier time. Consequently, our results revealed that scholars have, over the past two decades, become more likely to pay attention to therapeutics that have great advantages of potential clinical application. Among them, cardiac resynchronization therapy pacing (CRT-P), ICD, and RFCA play pivotal roles. Numerous landmark clinical trials, guidelines, and preclinical studies have confirmed the survival benefit of subcutaneous and transvenous ICD therapy for the primary and secondary prevention of SCD among candidates for various cardiovascular comorbidities and complications (2, 3, 7, 28, 29). Similarly, CRT-P has also long been included in international clinical guidelines as a fundamental and proven efficacious treatment mainly owing to its modification of the natural history of pathologic changes in patients with heart failure and electrical dyssynchrony and incremental improvement of patient symptoms and survival (28, 30, 31). However, the combined device of CRT-P and ICD, called a CRT-D, remains extremely controversial in terms of a number of factors including the appropriate population, success rates of implantation, risk of major complications, cost and cost-effectiveness, and indications for therapeutic use. Larger, multicenter, and high-quality RCTs should be conducted to address this issue (32–35). Unlike the former two, RFCA targets clinical arrhythmias to prevent recurrence and has a broader spectrum of indications, including different sites of origin of arrhythmogenesis. Furthermore, percutaneous catheter ablation has been considered to be a consistently effective treatment for recurrent VT and is performed with increasing frequency. Nevertheless, many unanswered questions remain, and challenges in the current era should be investigated and addressed by high-quality and large-scale clinical trials, such as the optimal timing for VT ablation, ablation in hemodynamically unstable and non-ischemic VT, and lack of emerging technologies (2, 3, 7, 8, 36, 37).

Compared with the keywords co-occurrence and analysis, the timeline views of the co-citation cluster and burst detection analyses more closely exemplify the changing trends of scientific hot topics in this field. As outlined previously, there has been a sudden shift from pathogenesis to clinical aspects in relevant VA studies since 2013, especially the attention given to clinical guidelines and evidence-based medicine. Indeed, constantly summarized expert consensus statements and updated clinical guidelines by authoritative scientific societies are crucial to help inform and guide clinical decision making for patients with VA. Theoretically, high-quality evidence-informed clinical guidelines are widely cited and drawn on. Therefore, the citation pattern and bibliometric distribution were naturally greatly impacted, which was also confirmed by the analysis results. However, a large amount of high-quality evidence for curative options is still lacking, although the use of these treatments has been repeatedly and highly recommended in many versions of VA guidelines.

Taken together with our results from the analysis of hot topics, one intervention strategy that attracted our attention is AAD. Although it did not appear in any of our results—either as a keyword in the co-occurrence and burst detection analyses or as a cluster label in the co-citation network analysis—it is still recommended because of its capability to control arrhythmias and improve symptoms. Moreover, as previously mentioned, evidence for AAD as an effective medication for VA-induced SCD and safe use remains limited and largely controversial (2, 3, 8, 9, 38). Even so, AAD medications have failed to become the primary concern to researchers compared with the widespread availability and effectiveness of the star therapy techniques mentioned in the previous paragraph. Importantly, it is essential for researchers and scholars to understand the mechanisms of action of antiarrhythmic drugs, and arrhythmogenesis is only a preliminary step in the appropriate selection of an AAD medication. Consequently, as well as higher-quality RCTs and meta-analyses focusing on mortality benefit and careful risk/benefit analysis with the chronic use of AAD (except beta-blockers) being needed, future studies should pay attention to its narrow therapeutic window, side effects, and proarrhythmic effects to overcome current clinical challenges and develop more rational applications.

Overall, the field has reached relative maturity and major scientific and academic breakthroughs are difficult to achieve in the near term unless landmark technological innovations are available. Nevertheless, this does not mean that the existing research results and evidence are adequate, and there is no room for further improvement. More creative experimental approaches and research ideas are required. An excellent paradigm for this is the recent work by Salvatore R. Aiello et al., whose experimental design and protocol were ingenious and clinically meaningful. This comparative study combined a large animal experiment with a clinical guideline-driven resuscitation protocol, and positive results were obtained, although the research topic was focused but not novel (i.e., AMSA) (39). More importantly, the starting point of this experimental design offers a significant benefit and valuable reference to researchers in this field.

## Conclusion

Our analysis of 6897 papers on VA-related research using the graphs and charts formed by bibliometric methods (i.e., CiteSpace, VOSviewer, the bibliometrix R package, and a bibliometric online analysis platform) demonstrated that the understanding of pathophysiological progression, effective intervention, and improvement in disease prognosis has advanced markedly over the past two decades, with relevant research deepening and being more fruitful and comprehensive. The findings of this study can assist researchers, scientists, and medical staff with more directional topic selection and experimental design schemes by offering a comprehensive picture of VA research and providing collective and constructive information.

To the best of our knowledge, this is the first bibliometric study of VA-related research activities. However, this study has the following major limitations. First, the data were only collected from the WoSCC database, which may have resulted in some selection bias. Second, some of the articles’ keywords and phrases as well as the cluster labels in the results appeared more than once because of the number of synonyms and different expressions. Finally, owing to space limitations, only a small proportion of the extracted cluster labels and keywords were discussed and only the first author in the clinical guidelines was shown in the tables presented in this paper. Nevertheless, the results obtained in this study were reliable and valid.

As research on VA is increasing significantly, the results of this study will change over time. Thus, this research requires constant updating in the coming years. Our team has provided an in-depth analysis and insights into the current research status. Although VA is relatively established and has made significant achievements, more reliable evidence (especially high-quality prospective studies) is needed to bridge the gap in evidence-based medicine; Moreover, wider adoption of social media and more novel ideas are necessary to develop and prompt popularization for the field further.

## Data availability statement

The original contributions presented in this study are included in the article/Supplementary material, further inquiries can be directed to the corresponding author.

## Ethics statement

Ethical review and approval was not required for this study in accordance with the local legislation and institutional requirements. Written informed consent was not required for this study in accordance with the local legislation and institutional requirements.

## Author contributions

XL, QY, and CZ contributed to the conception of the study, supervised the manuscript, and finalized the manuscript. TJ and SW performed the experiment, analyzed most of the data, and wrote the initial draft of the manuscript. GL contributed by refining the ideas and carrying out additional analyses. All authors contributed to the article and approved the submitted version.

## Footnotes

1
http://bibliometric.com/

2
http://www.bibliometrix.org

## References

[1] Olson EN. A decade of discoveries in cardiac biology. *Nat Med.* (2004) 10:467–74. 10.1038/nm0504-467 15122248

[2] Al-Khatib SM Stevenson WG Ackerman MJ Bryant WJ Callans DJ Curtis AB 2017 AHA/ACC/HRS Guideline for management of patients with ventricular arrhythmias and the prevention of sudden cardiac death: a report of the American College of Cardiology/American Heart Association Task Force on Clinical Practice Guidelines and the Heart Rhythm Society. *J Am Coll Cardiol.* (2018) 72:e91–220. 29097296 10.1016/j.jacc.2017.10.054

[3] John RM Tedrow UB Koplan BA Albert CM Epstein LM Sweeney MO Ventricular arrhythmias and sudden cardiac death. *Lancet.* (2012) 380:1520–9. 10.1016/S0140-6736(12)61413-5 23101719

[4] McElwee SK Velasco A Doppalapudi H. Mechanisms of sudden cardiac death. *J Nucl Cardiol.* (2016) 23:1368–79. 10.1007/s12350-016-0600-6 27457531

[5] Veeraraghavan R Gourdie RG Poelzing S. Mechanisms of cardiac conduction: a history of revisions. *Am J Physiol Heart Circ Physiol.* (2014) 306:H619–27. 10.1152/ajpheart.00760.2013 24414064 PMC3949060

[6] Kuriachan VP Sumner GL Mitchell LB. Sudden cardiac death. *Curr Probl Cardiol.* (2015) 40:133–200. 10.1016/j.cpcardiol.2015.01.002 25813838

[7] Roberts-Thomson KC Lau DH Sanders P. The diagnosis and management of ventricular arrhythmias. *Nat Rev Cardiol.* (2011) 8:311–21. 10.1038/nrcardio.2011.15 21343901

[8] Deyell MW AbdelWahab A Angaran P Essebag V Glover B Gula LJ 2020 Canadian Cardiovascular Society/Canadian Heart Rhythm Society position statement on the management of ventricular tachycardia and fibrillation in patients with structural heart disease. *Can J Cardiol.* (2020) 36:822–36. 10.1016/j.cjca.2020.04.004 32536373

[9] Tisdale JE Chung MK Campbell KB Hammadah M Joglar JA Leclerc J Drug-induced arrhythmias: a scientific statement from the American Heart Association. *Circulation.* (2020) 142:e214–33. 10.1161/CIR.0000000000000905 32929996

[10] Thompson DF Walker CK. A descriptive and historical review of bibliometrics with applications to medical sciences. *Pharmacotherapy.* (2015) 35:551–9. 10.1002/phar.1586 25940769

[11] Aria M Cuccurullo C. bibliometrix: an R-tool for comprehensive science mapping analysis. *J Informetr.* (2017) 11:959–75. 10.1016/j.joi.2017.08.007

[12] Lindsay JM. PlumX from plum analytics: not just altmetrics. *J Electron Resour Med Libr.* (2016) 13:8–17. 10.1080/15424065.2016.1142836

[13] Wong EY Vital SM. PlumX: a tool to showcase academic profile and distinction. *Digit Libr Perspect.* (2017) 33:305–13. 10.1108/DLP-12-2016-0047

[14] Borner K Chen CM Boyack KW. Visualizing knowledge domains. *Ann Rev Inform Sci Technol.* (2003) 37:179–255. 10.1002/aris.1440370106

[15] Hirsch JE. Does the H index have predictive power? *Proc Natl Acad Sci USA.* (2007) 104:19193–8. 10.1073/pnas.0707962104 18040045 PMC2148266

[16] Brookes BC. Bradford’s law and the bibliography of science. *Nature.* (1969) 224:953–6. 10.1038/224953a0 4902657

[17] Chen CM. Science mapping: a systematic review of the literature. *J Data Inform Sci.* (2017) 2:1–40. 10.1515/jdis-2017-0006

[18] Maron BJ Estes NAIII. Commotio cordis. *N Engl J Med.* (2010) 362:917–27. 10.1056/NEJMra0910111 20220186

[19] Surawicz B. Brief history of cardiac arrhythmias since the end of the nineteenth century: part I. *J Cardiovasc Electrophysiol.* (2003) 14:1365–71. 10.1046/j.1540-8167.2003.03320.x 14678115

[20] Ortega JL. The presence of academic journals on Twitter and its relationship with dissemination (tweets) and research impact (citations). *Aslib J Inform Manag.* (2017) 69:674–87. 10.1108/AJIM-02-2017-0055

[21] Bougioukas KI Bouras EC Avgerinos KI Dardavessis T Haidich AB. How to keep up to date with medical information using web-based resources: a systematised review and narrative synthesis. *Health Info Libr J.* (2020) 37:254–92. 10.1111/hir.12318 32691960

[22] Strielkowski W Chigisheva O. Research functionality and academic publishing: gaming with altmetrics in the digital age. *Econ Sociol.* (2018) 11:306–16. 10.14254/2071-789X.2018/11-4/20

[23] Jia T Luo C Wang S Wang Z Lu X Yang Q Emerging trends and hot topics in cardiopulmonary resuscitation research: a bibliometric analysis from 2010 to 2019. *Med Sci Monit.* (2020) 26:e926815. 10.12659/MSM.926815 33166272 PMC7664159

[24] Brown CG Dzwonczyk R. Signal analysis of the human electrocardiogram during ventricular fibrillation: frequency and amplitude parameters as predictors of successful countershock. *Ann Emerg Med.* (1996) 27:184–8. 10.1016/S0196-0644(96)70346-3 8629749

[25] Marn-Pernat A Weil MH Tang W Pernat A Bisera J. Optimizing timing of ventricular defibrillation. *Crit Care Med.* (2001) 29:2360–5. 10.1097/00003246-200112000-00019 11801840

[26] Young C Bisera J Gehman S Snyder D Tang W Weil MH. Amplitude spectrum area: measuring the probability of successful defibrillation as applied to human data. *Crit Care Med.* (2004) 32:S356–8. 10.1097/01.CCM.0000134353.55378.88 15508659

[27] Nakagawa Y Amino M Inokuchi S Hayashi S Wakabayashi T Noda T. Novel CPR system that predicts return of spontaneous circulation from amplitude spectral area before electric shock in ventricular fibrillation. *Resuscitation.* (2017) 113:8–12. 10.1016/j.resuscitation.2016.12.025 28104427

[28] Steffen MM Osborn JS Cutler MJ. Cardiac implantable electronic device therapy: permanent pacemakers, implantable cardioverter defibrillators, and cardiac resynchronization devices. *Med Clin North Am.* (2019) 103:931–43. 31378335 10.1016/j.mcna.2019.04.005

[29] Kusumoto FM Bailey KR Chaouki AS Deshmukh AJ Gautam S Kim RJ Systematic review for the 2017 AHA/ACC/HRS guideline for management of patients with ventricular arrhythmias and the prevention of sudden cardiac death: a report of the American College of Cardiology/American Heart Association Task Force on Clinical Practice Guidelines and the Heart Rhythm Society. *J Am Coll Cardiol.* (2018) 72:1653–76. 10.1161/CIR.0000000000000550 29097297

[30] Chatterjee NA Singh JP. Cardiac resynchronization therapy: past, present, and future. *Heart Fail Clin.* (2015) 11:287–303. 10.1016/j.hfc.2014.12.007 25834976

[31] Boriani G Nesti M Ziacchi M Padeletti L. Cardiac resynchronization therapy: an overview on guidelines. *Heart Fail Clin.* (2017) 13:117–37. 10.1016/j.hfc.2016.07.010 27886918

[32] Normand C Linde C Bogale N Blomström-Lundqvist C Auricchio A Stellbrink C Cardiac resynchronization therapy pacemaker or cardiac resynchronization therapy defibrillator: what determines the choice? Findings from the ESC CRT Survey II. *Europace.* (2019) 21:918–27. 10.1093/europace/euz002 31157387

[33] Daubert JC Martins R Leclercq C. Why we have to use cardiac resynchronization therapy-pacemaker more. *Heart Fail Clin.* (2017) 13:153–64. 10.1016/j.hfc.2016.07.012 27886920

[34] Levy WC. Should nonischemic crt candidates receive CRT-P or CRT-D? *J Am Coll Cardiol.* (2017) 69:1679–82. 10.1016/j.jacc.2017.01.044 28359512

[35] Jiang M He B Zhang Q. Comparison of CRT and CRT-D in heart failure: systematic review of controlled trials. *Int J Cardiol.* (2012) 158:39–45. 10.1016/j.ijcard.2010.12.091 21262545

[36] Cronin EM Bogun FM Maury P Peichl P Chen M Namboodiri N 2019 HRS/EHRA/APHRS/LAHRS expert consensus statement on catheter ablation of ventricular arrhythmias. *Europace.* (2019) 21:1143–4. 10.1093/europace/euz202 31075787 PMC7967791

[37] Guandalini GS Liang JJ Marchlinski FE. Ventricular tachycardia ablation: past, present, and future perspectives. *JACC Clin Electrophysiol.* (2019) 5:1363–83. 10.1016/j.jacep.2019.09.015 31857035

[38] Mankad P Kalahasty G. Antiarrhythmic drugs: risks and benefits. *Med Clin North Am.* (2019) 103:821–34. 10.1016/j.mcna.2019.05.004 31378328

[39] Aiello SR Mendelson JB Baetiong A Radhakrishnan J Gazmuri RJ. Targeted delivery of electrical shocks and epinephrine, guided by ventricular fibrillation amplitude spectral area, reduces electrical and adrenergic myocardial burden, improving survival in swine. *J Am Heart Assoc.* (2021) 10:e023956. 10.1161/JAHA.121.023956 34743550 PMC9075377

