# Supplementary material for: Mapping the research trends and hot topics of ventricular arrhythmia: A bibliometric analysis from 2001 to 2020
Source: Front Cardiovasc Med. 2022 Oct 20;9:856695. doi: 10.3389/fcvm.2022.856695 (PMC9631785; doi:10.3389/fcvm.2022.856695)
Supplement: Supplementary file 1 [file Table_1.docx]

| Bibliometric parameters | Definitions | Utility in bibliometric evaluation |
| --- | --- | --- |
| Betweenness centrality (centrality) | The frequency of a node as a mediator variable in a certain network | A centrality measure of a node (e.g., authors, affiliations and articles) within a certain bibliometric network |
| h-index | An author/country published “h” papers, each of which has been cited in other journals at least “h” times | A mixed quantitative measure of an author/country’s high-citations |
| m-index | Calculated from the h-index weighted for an author’s career duration in years | A highly representative derived measure of an individual’s scientific achievement |
| Cited half-life (CHL) | The time (in years) required for the articles to reach nearly half of the current citations received | A sensitive measure of timeliness and aging for updates in important fields of a journal or publication |
| PlumX metrics | Assessment of the impact of the scientific research on common social media | A composite measure of browse records for scientific researches or articles on on-academic platforms |
| Articles fractionalized frequency (AFF) | $frac Freq\left( AU_{x} \right)=\sum_{h\in AU_{x}} \frac{1}{n. of Co\text{-}authors (h)}$ | A quantitative measure of contribution rate of authors to their academic paper |

**Supplementary table 1** Definitions of major bibliometric terms, metrics and indicators in our research.

$AU_{x}$ is the set of documents that are co-authored by the author $x$; $h$ is a document included in $AU_{x}$
